# New hybrid multiplanar cone beam computed tomography-laser-fluoroscopic-guided approach in cochlear implant surgery

**DOI:** 10.1007/s11548-022-02703-2

**Published:** 2022-07-11

**Authors:** Stephan Waldeck, Sandra Schmidt, Christian von Falck, René Chapot, Marc Brockmann, Daniel Overhoff

**Affiliations:** 1 Department of Diagnostic and Interventional Radiology and Neuroradiology, Bundeswehr Central Hospital Koblenz, Rübenacher Straße 170, 56072 Koblenz, Germany; 2 Institute of Neuroradiology, University Medical Centre Johannes Gutenberg University Mainz, Mainz, Langenbeckstraße 1, 55131 Germany; 3 Department of ENT Surgery, Bundeswehr Central Hospital Koblenz, Rübenacher Straße 170, 56072 Koblenz, Germany; 4 Institute of Diagnostic and Interventional Radiology, Hannover Medical School, Carl-Neuberg-Straße 1, 30625 Hanover, Germany; 5 Department of Neuroradiology, Alfried Krupp Krankenhaus, Alfried-Krupp-Strasse 21, 45131 Essen, Germany; 6 Department of Radiology and Nuclear Medicine, University Medical Centre Mannheim, Medical Faculty Mannheim, Heidelberg University, Mannheim, Germany

**Keywords:** Cochlea implant, CBCT, Hybrid laser-fluoroscopic, Intraoperative imaging

## Abstract

**Purpose:**

Cochlea implant surgery with proper positioning of the cochlear electrode can be challenging. Intraoperative real-time hybrid laser-fluoroscopic-guided navigation based on a multiplanar cone beam computed tomography (CBCT) dataset opens up the opportunity to immediate radiological control of primary electrode misalignments and offering new insights into the cochlea electrode insertion routes and favorable cochlear implant-insertion angle.

**Methods:**

In this retrospective study, 50 cases (29 males, 18 females) of conventional electrode implantation (without intraoperative image control; group A) and nine cases (7 males, 2 females) of CBCT-laser-fluoroscopic-guided surgery (group B) were included in the present study. CBCT-laser-guided surgery under real-time fluoroscopic control was conducted using an intraoperative C-arm CBCT. All patients received preoperative cross-sectional imaging (CT and MRI), in which cochlear malformation could be excluded. Postoperatively, we looked for electrode misplacements.

**Results:**

In group A, electrode misalignment was detected postoperatively in 14 of 50 cases (28.0%). In group B, primary electrode misalignment was detected intraoperatively in two patients (22.2%). In both patients, the misalignments were corrected in the same session. The comparison of cochlear insertion angles showed significant differences. Group A: 47.5 ± 2.6° (actual conventional surgery) vs 17.6 ± 2.8° (theoretical CBCT-laser-fluoroscopic-guided surgery) *P* < 0.001. Group A vs group B: 47.5 ± 2.6° (actual conventional surgery; Group A) vs 17.9 ± 2.5° (actual CBCT-laser-fluoroscopic-guided surgery; Group B) *P* < 0.001.

**Conclusion:**

We consider that an intraoperative hybrid CBCT-laser-fluoroscopic-controlled approach in cochlear implant surgery using a C-arm CT can be beneficial, because electrode misalignments can be reduced and if it does occur, remedied in the same surgical session.

## Introduction

Cochlear implant surgery is constantly evolving. Not only preoperative diagnostics and planning are crucial for a good result, but also more often intraoperative image-guided electrode navigation. For optimal hearing results in cochlear implant surgery, the implanted electrode should be located completely within the scala tympani.

The theoretically achievable insertion depth is not often achievable in practice for many patients. In addition, accidental scale shift into the scala vestibuli is to be avoided [1]. Equally, electrode tip fold-over and other dislocations of the electrode array affect the correct functioning of the implant negatively, leading to complete dysfunction in the worst case [2–4].

The importance of accurate navigation in cochlear implantation has been widely recognized. Previously, alternative methods like the three-dimensional CT-based method for preoperative imaging have been proposed [5]; however, they do not lend real-time guidance to the surgeon during surgery. In past years, electrophysiological measurements [6], fluoroscopy [7], and transorbital or head X-ray [8, 9] have been available during surgery in a routine setting, and they have shown clear benefits with regard to correct electrode placement. With timely use of intraoperative radiological diagnostics, the need for revisions can be largely obviated [10].

We have developed a hybrid laser-fluoroscopic-guided CBCT approach to resolve the above challenging issues in the cochlear implantation. We believe that the system can support the planning and execution of the access approach to the round window, as well as the optimization of the electrode insertion angle and intraoperative 3D electrode array monitoring in the cochlea. The aim of study here is to evaluate incidence of electrode misalignment and electrode insertion angle into the cochlear in the hybrid CBCT-laser-fluoroscopic-guided cochlear implant surgery in comparison of those in the conventional approach.

## Materials and methods

The local ethics committee approved this retrospective study (Nr: 2021-15,837).

### Patient cohort

The study cohort consisted of 50 cases (47 patients) of conventional cochlear implantation and nine cases (nine patients) of cochlear implantation by CT-laser-guided surgery.

Conventional surgery group A: Patients’ age in the conventional surgery cohort ranged from 9 to 80 years at the time of surgery. Nineteen patients were female, and 28 were male. Implantations were conducted between June 2013 and January 2018. Twenty-one right-sided and 29 left-sided implantations were performed.

Hybrid CBCT-laser-fluoroscopic-guided surgery group B: Patients’ ages in the laser-guided surgery cohort ranged from 19 to 64 years at the time of surgery. Two patients were female, and seven patients were male. Implantations were conducted between August 2017 and March 2018. Four right-sided and five left-sided implantations were performed.

### Examination technique

The surgical approach in group B was conducted using a novel robotically controlled C-arm-system for flexible intraoperative imaging and navigation, and the ARTIS Pheno (Siemens Healthineers, Forchheim, Germany) CBCT imaging was performed before and after cochlear surgery.

Intraoperatively, the primary CBCT reconstructions were preformed directly after anesthetic induction and head fixation (with self-adhesive fixation tapes) using a workstation (SyngoVia V10B, Siemens Healthineers, Forchheim, Germany).

**Fig. 1:**
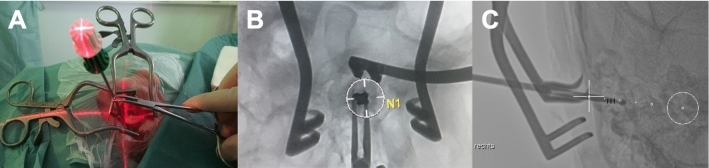
Illustration of the hybrid CBCT- laser-fluoroscopic-guided procedure. **A** CBCT-laser-fluoroscopic-guided insertion of the drill channel. Shown is the drill with the centered laservization for the access path determined by CBCT **B** Corresponding fluoroscopic bullseye view of the navigated insertion. Centered in the white circle the drill is pictured. **C** Fluoroscopic second view from another viewing angle of the navigated insertion. The drill can be delimited here in its longitudinal extension

**Fig. 2:**
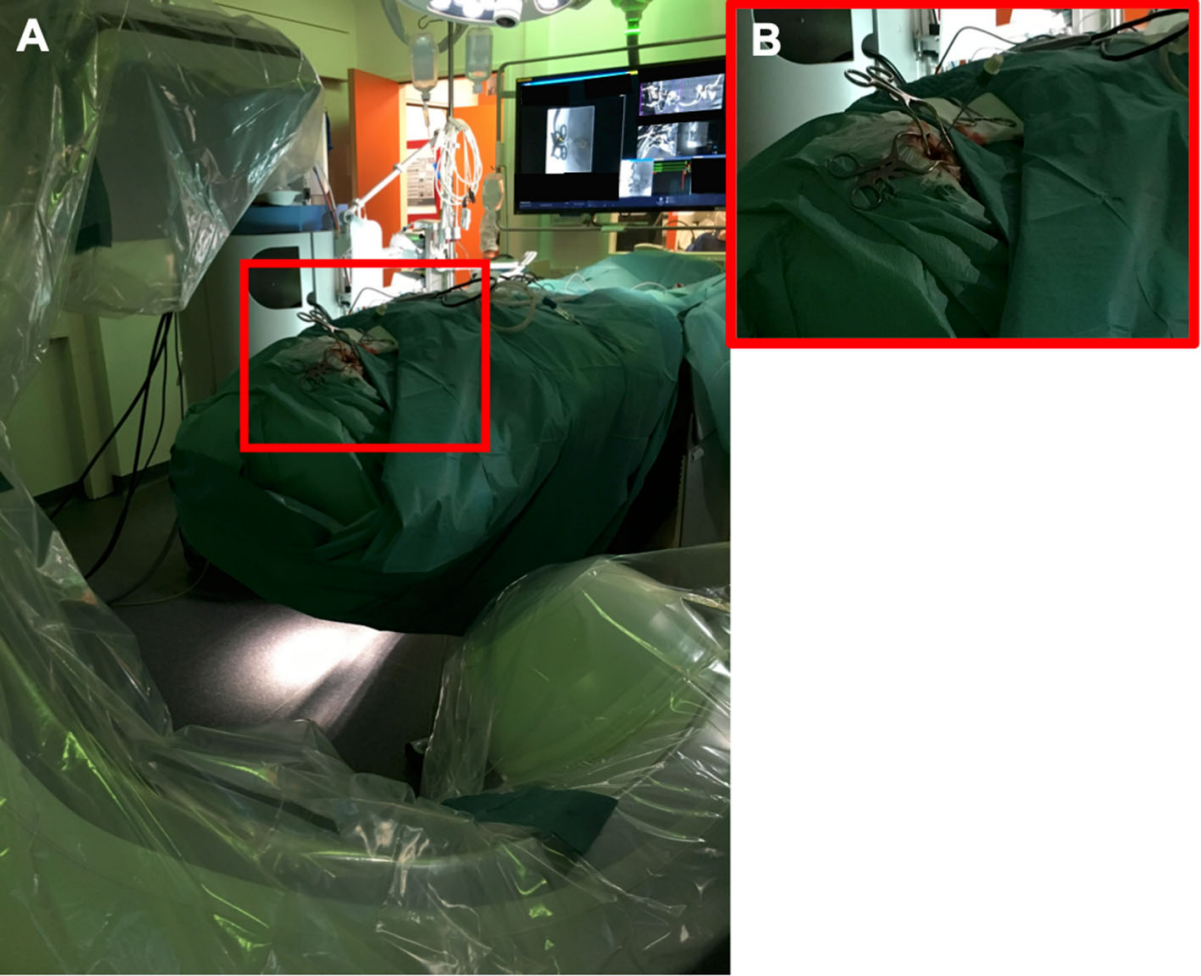
Surgical setup. Visualization of the surgical setup. **A** Operating field with mastoidectomy centered between detector (left upper corner) and X-ray tube (right lower corner). **B** enlarged view of the operating field

Based on these multiplanar reconstructions, the surgical approach was performed by mastoidectomy in the usual way. The optimized multiplanar insertion angle of the electrode array into the cochlea was also planned and determined jointly by an experienced ear surgeon and a neuroradiologist in an interdisciplinary manner. The jointly verified electrode insertion angle was drilled with a thin drill channel based on the multiplanar image data set using the SyngoVia “needle guidance” application in the appropriate automatically calculated fluoroscopic angles (e.g., bulls eye view) and additional laser navigated projection for the surgeon (Fig. 1). The general setup is shown in Fig. 2. The application visualized the insertion angle in a double oblique reconstruction, which showed the cochlear turns in relation to the round window membrane.

In addition, the facial nerve was controlled by means of neuromonitoring. Corresponding to the angulation of the drill channel, the cochlear electrode was inserted into the round window under real-time fluoroscopy. Electrodes from MED-El® (Starnberg, Germany) were used for all implantations. Access was through the round window in both groups. No intraoperative complications occurred in both groups.

After complete implantation of the electrode array, a second CBCT scan was immediately performed to check the intratympanic electrode position.

The CBCT (ARTIS Pheno, Siemens Healthineers, Forchheim, Germany) scan used a 30 × 40 detector with a 3D volume size of 13 × 9.3 inches and automated tube voltage selection (CARE kV). The quality reference tube voltage was 90 kV. Intraoperative laser-fluoroscopic imaging was based on three automatically calculated projections (needle guidance application) with microfocus and overlaid navigation paths (Fig. 1).

Images were obtained using a rotation time = 4 s, section thickness = 0.25 mm, and section interval = 0.1 mm. Reconstruction was performed using the Hr60 kernel.

Postoperative CBCT controls were performed immediately after cochlear implantation with the surgical access route not yet closed. This allowed immediate correction of electrode misalignments.

In group A, the postoperative radiological assessment was conducted by computed tomography (MSCT) in 32 cases and by cone beam CT in 18 cases.

The MSCT scan used the Somatom Force (dual-source 192-dectetor row scanner; Siemens Healthineers, Forchheim, Germany) with single-energy automated tube voltage selection (CARE kV). The quality reference tube voltage was 120 kV, providing an acquisition of 100 kV and a tube current of 375 mAs.

Images were obtained using a beam collimation = 0.5 mm, rotation time = 0.25 s, FOV = 240 mm, section thickness = 0.5 mm, section interval = 0.25 mm, and pitch = 0.8. Reconstruction was performed using the Hr60 kernel.

The cone beam CT was performed by the CBCT 3D Accuitomo 170 (J Morita Mfg Corp., Kyoto, Japan) using 90-kV tube voltage and 5-mA current, with a high-resolution mode with a rotation of 180 degrees. A voxel size of 0.125 mm and an ROI of 80 × 80 × 80 mm are observed. Images were reconstructed with filtered back-projection using the G_001 reconstruction algorithm.

### Image evaluation

All obtained images in both groups were reconstructed using a workstation (SyngoVia V10B, Siemens Healthineers, Forchheim, Germany) and examined by two experienced neuroradiologists.

First, the electrode array positions were assessed in the cochlear with respect to the presence of misalignments (Table 1).

**Table 1:** Diagnoses of CI misalignments

Scalar shift	10
Fold-over	1
Electrode buckling	1
Primary scala vestibuli insertion	2
Extra-cochlear electrode placement	2

Overview of occurrences of improper electrode placement and diagnosis in both groups

**Fig. 3:**
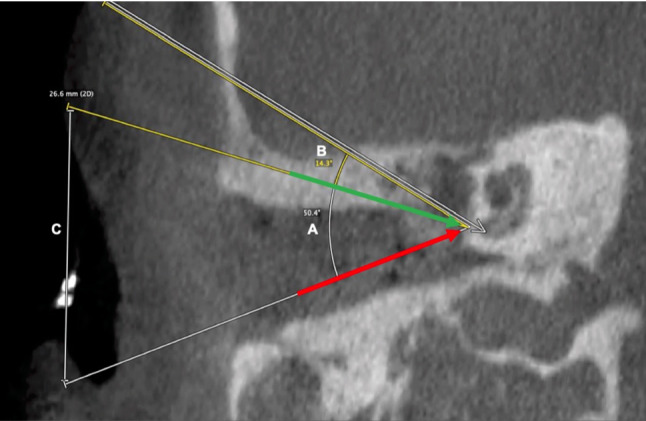
Visualization of the different insertion angles (exemplary representation of a patient of conventional surgery group A). White arrow represents the optimal insertion vector (theoretical; through the middle cranial fossa). Green arrow represents the theoretical hybrid CBCT-laser-fluoroscopic-guided insertion vector. Red arrow represents the actual surgical insertion vector. (A) angle of deviation of the conventional surgery vector from the optimal insertion vector (angle alpha). (B) Angle of deviation of the hybrid CBCT-laser-fluoroscopic-guided insertion vector from the optimal insertion vector (angle beta). (C) Cutaneous distance between actual surgical vector and the theoretical hybrid CBCT-laser-fluoroscopic-guided vector

Second, we determined the optimal insertion vector aligned with the basal turn of the cochlea/scala tympani. Due to anatomical conditions, this angle would mostly lead inoperably through the middle cranial fossa. The deviations (angle alpha and beta) in our study are calculated in relation to this optimal insertion vector (Fig. 3).

Angle alpha describes the degree of deviation of the conventional surgery cochlear electrode insertion from the optimal insertion vector. Angle beta represents the degree of deviation from the hybrid CBCT-laser-fluoroscopic-guided insertion from optimal insertion vector. (Table 2).

**Table 2:** Comparison of different insertion angels

Conventional surgery (actual)	Mean insertion angle ± SD (degree)		
	47.5 ± 2.6	47.5 ± 2.6	
CBCT-laser-guided surgery (theoretical)	17.6 ± 2.8		17.6 ± 2.8
CBCT-laser-guided surgery (actual)		17.9 ± 2.5°	17.9 ± 2.5°
*P*-value	*P* < 0.001	*P* < 0.001	*P* = 0.763

Comparison of actual insertion angle from conventional surgery group, theoretical insertion angle of hybrid CBCT-laser-fluoroscopic-guided surgery method (postoperative control CBCT) and actual insertion angle from hybrid CBCT-laser-fluoroscopic-guided surgery group

### Statistical analysis

The statistical calculations were performed using SPSS (IBM SPSS Statistics, version 20.0 for Macintosh; SPSS, Inc., Chicago, IL, USA).

All continuous variables are expressed as arithmetic mean ± standard deviation (SD). A significance level of 5% was used.

The different insertion angles were evaluated with paired t test and unpaired t test. Normal distribution of the data sets was analyzed with Kolmogorov–Smirnov test. Chi-square test was used to analyze the frequencies of electrode misalignment for the different surgical methods.

## Results

The operation time ranged from 82 to 124 min (mean 106 ± 12) in group A, and from 175 to 237 min in the group B (mean 198 ± 19 min; vs. group A, *P* < 0.001, t test).

### Cochlear electrode array misalignment

**Fig. 4:**
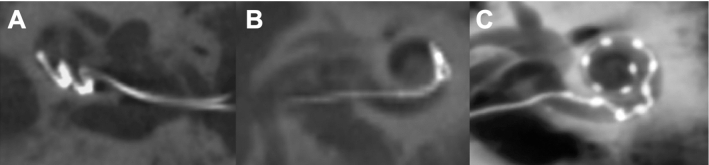
Examples of different types of CI electrode misalignments. **A** buckling of the electrode array (MED-EL® Flex 24). **B** fold-over of the electrode array (MED-EL® Flex 20). **C** accidental scale shift of the electrode array (MED-EL® Flex 26)

The incidence of misalignment of electrodes yielded no statistical significance comparing group A and group B (*P* = 0.720). In the group A, electrode array misalignment was detected postoperatively in 14 of 50 cases (28.0%); ten cases needed a revision. In four cases, the patients experienced a subjective hearing improvement after implantation, so the patients declined revision surgery. In one patient, the electrode array was misaligned initially as well as in the revision. In ten cases, a primary displacement from scala tympani to scala vestibuli was detected; in two cases, such a misalignment was preceded by fold-over (one case) or buckling (one case) of the electrode array. In one case, vestibular displacement was accompanied by complete dislocation of the electrode. In two other cases, the cochlear implant and the electrodes, respectively, were inserted incompletely (Fig. 4). In this group B, primary electrode array misalignment was detected intraoperatively in two patients (22.2%): In one of them, a primary misalignment to the scala vestibuli most likely caused by considerable sclerosis of the cochlea occurred, in the other, a primary displacement to the sacculus was detected. In both, the displacement was corrected in the same session. The follow-up scans did not reveal any displacements (Table 1).

### Cochlear insertion angles

**Fig. 5:**
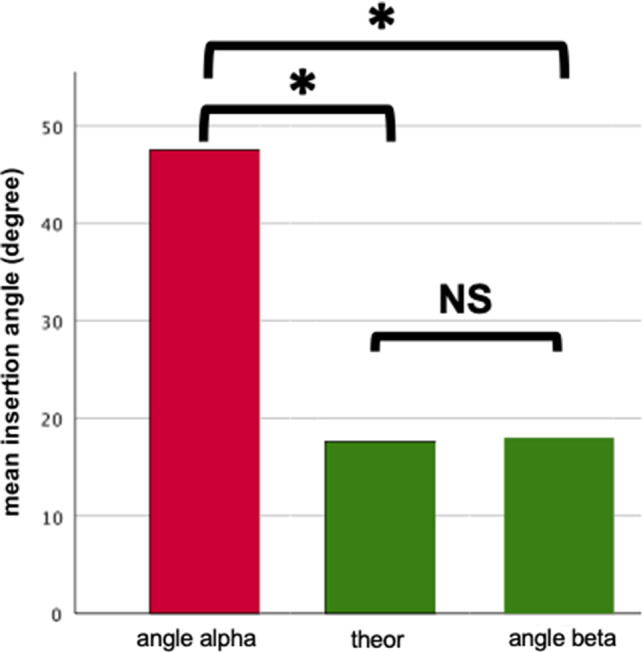
Bar charts of comparison of different insertion angels. Comparison of actual insertion angle from conventional surgery group (alpha angle) with theoretical insertion angle (theor) of hybrid CBCT-laser-fluoroscopic-guided surgery method (postoperative control CBCT) (*P* < 0.001). Comparison of actual insertion angle of conventional surgery group A with actual insertion angle from hybrid CBCT-laser-fluoroscopic-guided surgery (beta angle) (*P* < 0.001). Comparison of theoretical CBCT-laser-fluoroscopic-guided surgery insertion angle from conventional surgery group with actual insertion angle from hybrid CBCT-laser-fluoroscopic-guided surgery group B (*P* = 0.763). asterisks indicate significance, NS Nonsignificant

In group A, the actual angle of deviation from the optimal insertion vector was 47.5 ± 2.6 degrees (angle alpha). In the group B, the angle of deviation from the optimal insertion vector was 17.9 ± 2.5 degrees (angle beta). The angles of the both groups were significantly different (*P* < 0.001)(Fig. 5). Additionally, the theoretic possible CBCT-laser-guided insertion angle of group A was 17.6 ± 2.8 degrees, which is significant different from angle alpha (*P* < 0.001, t-test), but similar to angle beta (Fig. 5).

## Discussion

In our study, we analyzed a hybrid CBCT-laser-fluoroscopic-guided surgery. The study revealed that this procedure has the potential to improve outcomes even further than intraoperative radiology alone. An essential difference to the technique presented by Bárdosi et al. [11] is the real-time CBCT fluoroscopic laser navigation in our study.

This means that potential disadvantages such as a coregistration of a preoperatively prepared CT data set with the associated risk of potential false registration can be dispensed with. Given the success of navigation support in sinus surgery [11, 12], thoracoscopy [13, 14], lumbar spine surgery [15], and cardiac implantation of electronic devices [16], we evaluated its usefulness for the cochlear implantation.

The study demonstrates that the real-time CBCT fluoroscopic laser navigation can be possible and beneficial in cochlear implantation as well. In particular, the surgeon has the possibility of a real-time double check of his surgical access route to the round window membrane by means of laser navigation and, if necessary, supplementary corresponding navigated fluoroscopy to immediately detect deviations from the planned access route (Fig. 1). The likelihood for correct placement of the electrode in the scala tympani is the highest in a surgical round window approach [1]. The electrodes of all patients in this study were inserted through the round window approach. The difficulty of electrode implantation through the round window is dependent on the patient’s anatomy; a larger angle between the line connecting the leading edge of the facial nerve to the midpoint of the round window and the median sagittal line, which can be measured in preoperative CT scans, is associated with a greater difficulty in round window implantation [17]. Poor mastoid aeration and lower position of the tegmen are also associated with higher difficulty in accessing the round window [18]. The real-time CBCT fluoroscopic laser navigation is capable of detecting deviations from the correct angle of insertion during surgery.

Electrode malposition can also be detected during surgery because the advancement of electrode array is immediately visualized on the monitor. The incidence of electrode misalignment is reported to be below 3% in the literature [19, 20]. However, our clinical experience and the data in the present study suggest that the number of undetected cases is considerably higher, due to a lack of standardization in postoperative imaging [21]. This is also reflected in the fact that there is a wide range of misalignment rates especially for scalar translocation, which is the most common type of misalignment, between different studies with up to 54% scalar translocation rates [21]. The results of our study with 28.0% overall misalignment in group A and 22.2% overall misalignment in group B are in this range.

Electrode kinking was found in 2.3% of cases as an intraoperative complication in one study [19]. The tip fold-over, which was not immediately clinically identifiable, occurred in 1.98% of cases in a study with 303 cochlea implantations [20].

These previous reports suggest that early intraoperative detection of electrode misalignment and thus real-time CBCT fluoroscopic laser navigation can be beneficial. Only if, in conjunction with the impedance measurement and the surgical assessment, this seems necessary for an improvement of the hearing results. Intraoperative detection of electrode malposition allows the electrode position to be changed in the same session.

### Study limitations

Our study focused on intraoperative and postoperative electrode misalignment as primary outcome. Outcome analysis with respect to clinical parameters such as length of hospitalization, postoperative complications such as infection and audiometric evaluation for the different study cohorts were not the primary target of this study. These analyses need to be further investigated in larger long-term multicenter studies.

The patient cohort of group B is limited in numbers due to the proof of concept. Therefore, the incidence of misalignment of electrodes might be large even in group B. We have also not yet focused on optimizing the prolonged temporal procedures for group B. Further investigations about the merits of this navigated access route in comparison with the conventional access routes remain to be conducted in larger numbers.

We have also not yet focused on optimizing the prolonged temporal procedures for group B. Further investigations about the merits of this navigated access route in comparison with the conventional access routes remain to be conducted in larger numbers.

## Conclusions

Even if the comparison of both surgical procedures in our study did not lead to statistically significant different results, we consider that the hybrid CBCT-laser-fluoroscopic-guided surgical approach in cochlear implant surgery can be beneficial because electrode misalignments as one of the important causes of postoperative implant malfunction might be reduced.

In the present study, using hybrid CBCT-laser-fluoroscopic-guided surgery with an intraoperative C-arm CBCT, the authors were able to identify a new, optimized navigated access route into the cochlear for the electrode insertion with a steeper angle toward the round window, reducing the risk of electrode misalignment.

In addition, real-time multiplanar cross-sectional imaging allows immediate control of intracochlear electrode position and, if necessary, direct correction, and it reduces radiation dose by eliminating the need for additional postoperative CT scans.

## Abbreviations

CBCT: Cone beam computed tomography
CT: Computed tomography
mAs: Milliampere-second
MSCT: Multislice computed tomography
kV: kilovoltage

## References

[1] O'Connell BP, Hunter JB, Wanna GB (2016). The importance of electrode location in cochlear implantation. Laryngoscope Investig Otolaryngol.

[2] Qiu J, Chen Y, Tan P, Chen J, Han Y, Gao L, Lu Y, Du B (2011). Complications and clinical analysis of 416 consecutive cochlear implantations. Int J Pediatr Otorhinolaryngol.

[3] Sabban D, Parodi M, Blanchard M, Ettienne V, Rouillon I, Loundon N (2018). Intra-cochlear electrode tip fold-over. Cochlear Implants Int.

[4] Migirov L, Muchnik C, Kaplan-Neeman R, Kronenberg J (2006). Surgical and medical complications in paediatric cochlear implantation: a review of 300 cases. Cochlear Implants Int.

[5] Verbist BM, Joemai RM, Briaire JJ, Teeuwisse WM, Veldkamp WJ, Frijns JH (2010). Cochlear coordinates in regard to cochlear implantation: a clinically individually applicable 3 dimensional CT-based method. Otol Neurotol.

[6] Garaycochea O, Manrique-Huarte R, Manrique M (2020). Intra-operative radiological diagnosis of a tip roll-over electrode array displacement using fluoroscopy, when electrophysiological testing is normal: the importance of both techniques in cochlear implant surgery. Braz J Otorhinolaryngol.

[7] Viccaro M, Covelli E, De Seta E, Balsamo G, Filipo R (2009). The importance of intra-operative imaging during cochlear implant surgery. Cochlear Implants Int.

[8] Hassan AM, Patel R, Redleaf M (2015). Intra-operative skull X-ray for misdirection of the cochlear implant array into the vestibular labyrinth. J Laryngol Otol.

[9] Viccaro M, De Seta E, Covelli E, Marvaso V, Filipo R (2008). Another reason for intra-operative imaging during cochlear implantation. J Laryngol Otol.

[10] Jiang Y, Gu P, Li B, Gao X, Sun B, Song Y, Wang G, Yuan Y, Wang C, Liu M, Han D, Dai P (2017). Analysis and management of complications in a cohort of 1065 minimally invasive cochlear implantations. Otol Neurotol.

[11] Bárdosi Z, Plattner C, Özbek Y, Hofmann T, Milosavljevic S, Schartinger V, Freysinger W (2020). CIGuide: in situ augmented reality laser guidance. Int J Comput Assist Radiol Surg.

[12] Khan M, Kosmecki B, Reutter A, Ozbek C, Keeve E, Olze H (2012). A noncontact laser-guided system for endoscopic computer-assisted sinus surgery. Surg Innov.

[13] Hsieh MJ, Fang HY, Lin CC, Wen CT, Chen HW, Chao YK (2018). Single-stage localization and removal of small lung nodules through image-guided video-assisted thoracoscopic surgery. Eur J Cardiothorac Surg.

[14] Cheng YF, Chen HC, Ke PC, Hung WH, Cheng CY, Lin CH, Wang BY (2020). Image-guided video-assisted thoracoscopic surgery with Artis Pheno for pulmonary nodule resection. J Thorac Dis.

[15] Richter PH, Gebhard F, Salameh M, Schuetze K, Kraus M (2017). Feasibility of laser-guided percutaneous pedicle screw placement in the lumbar spine using a hybrid-OR. Int J Comput Assist Radiol Surg.

[16] Zardo P, Busk H, Hadem J, Baraki H, Kensah G, Kutschka I (2016). A novel video-assisted approach to excimer laser-guided cardiac implantable electronic devices lead extraction. Innovations (Phila).

[17] Xie L-H, Tang J, Miao W-J, Tang X-L, Li H, Tang A-Z (2018). Preoperative evaluation of cochlear implantation through the round window membrane in the facial recess using high-resolution computed tomography. Surg Radiol Anat.

[18] Park E, Amoodi H, Kuthubutheen J, Chen JM, Nedzelski JM, Lin VY (2015). Predictors of round window accessibility for adult cochlear implantation based on pre-operative CT scan: a prospective observational study. J Otolaryngol-Head Neck Surg.

[19] Hsieh HS, Wu CM, Zhuo MY, Yang CH, Hwang CF (2015). Intraoperative facial nerve monitoring during cochlear implant surgery: an observational study. Medicine (Baltimore).

[20] Zuniga MG, Rivas A, Hedley-Williams A, Gifford RH, Dwyer R, Dawant BM, Sunderhaus LW, Hovis KL, Wanna GB, Noble JH, Labadie RF (2017). Tip fold-over in cochlear implantation: case series. Otol Neurotol.

[21] Dong Y, Briaire JJ, Siebrecht M, Stronks HC, Frijns JHM (2021). Detection of translocation of cochlear implant electrode arrays by intracochlear impedance measurements. Ear Hear.

